# Metabolic Functions of G Protein-Coupled Receptors and β-Arrestin-Mediated Signaling Pathways in the Pathophysiology of Type 2 Diabetes and Obesity

**DOI:** 10.3389/fendo.2021.715877

**Published:** 2021-08-23

**Authors:** Camila Oliveira de Souza, Xuenan Sun, Dayoung Oh

**Affiliations:** Touchstone Diabetes Center, Department of Internal Medicine, University of Texas Southwestern Medical Center, Dallas, TX, United States

**Keywords:** GPCRs, β-arrestins, biased signaling, type 2 diabetes, obesity

## Abstract

Seven transmembrane receptors (7TMRs), often termed G protein-coupled receptors (GPCRs), are the most common target of therapeutic drugs used today. Many studies suggest that distinct members of the GPCR superfamily represent potential targets for the treatment of various metabolic disorders including obesity and type 2 diabetes (T2D). GPCRs typically activate different classes of heterotrimeric G proteins, which can be subgrouped into four major functional types: G_αs_, G_αi_, G_αq/11_, and G_12/13_, in response to agonist binding. Accumulating evidence suggests that GPCRs can also initiate β-arrestin-dependent, G protein-independent signaling. Thus, the physiological outcome of activating a certain GPCR in a particular tissue may also be modulated by β-arrestin-dependent, but G protein-independent signaling pathways. In this review, we will focus on the role of G protein- and β-arrestin-dependent signaling pathways in the development of obesity and T2D-related metabolic disorders.

## Introduction

Type 2 diabetes (T2D) is a complex, heterogeneous disease afflicting an increasing proportion of the population. In 2018, around 8.2% of the United States population had T2D (1). Insulin resistance is key to the pathogenesis of T2D, and obesity is the most common cause of insulin resistance in humans (2). As the worldwide prevalence of obesity is rising to epidemic proportions, a parallel epidemic of T2D is eminent (3). In most individuals, insulin resistance can be compensated by pancreatic β-cells through hyperinsulinemia. However, eventually β-cell dysfunction emerges and is characterized by a decrease in β-cell mass, as well as poor ability of β-cells to correctly secrete insulin in response to glucose. In this context, hyperinsulinemia is no longer able to compensate resulting in hyperglycemia and the development of T2D (2, 4, 5). Therefore, insulin resistance and lower insulin secretion are the two coexisting pathophysiological markers in most patients with T2D (2, 4, 5).

**Figure 1:**
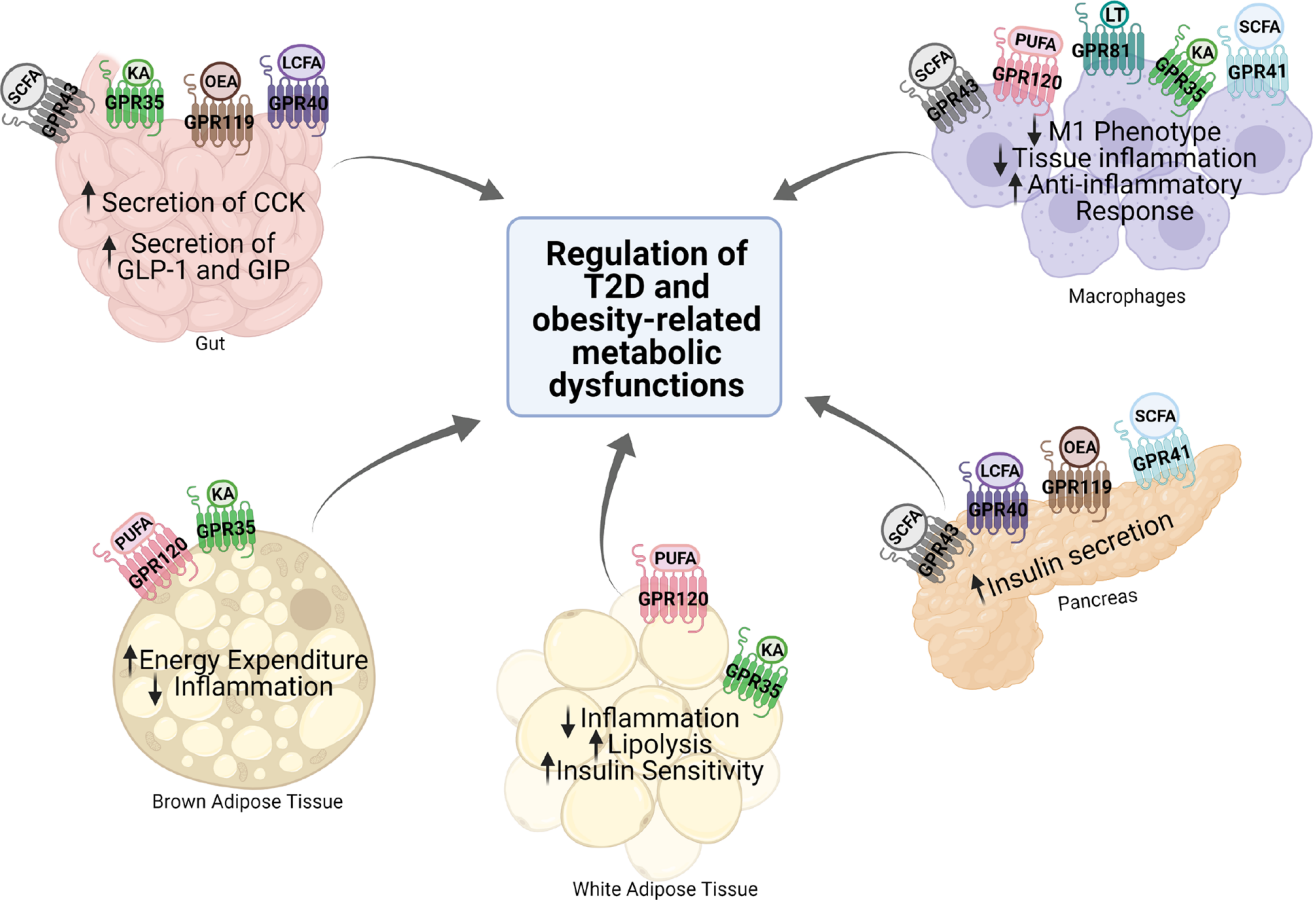
Expression and function of various GPCRs in metabolic tissues. Various GPCRs target insulin resistance, obesity, and T2D-related pathophysiology in the metabolic tissues. KA, kynurenic acid; LCFA, long-chain fatty acid; SCFA, short-chain fatty acid; OEA, oleylethanolamide; PUFA, polyunsaturated fatty acid; CCK, cholecystokinin; GLP-1, glucagon-like peptide 1; GIP, gastric inhibitory polypeptide.

**Figure 2:**
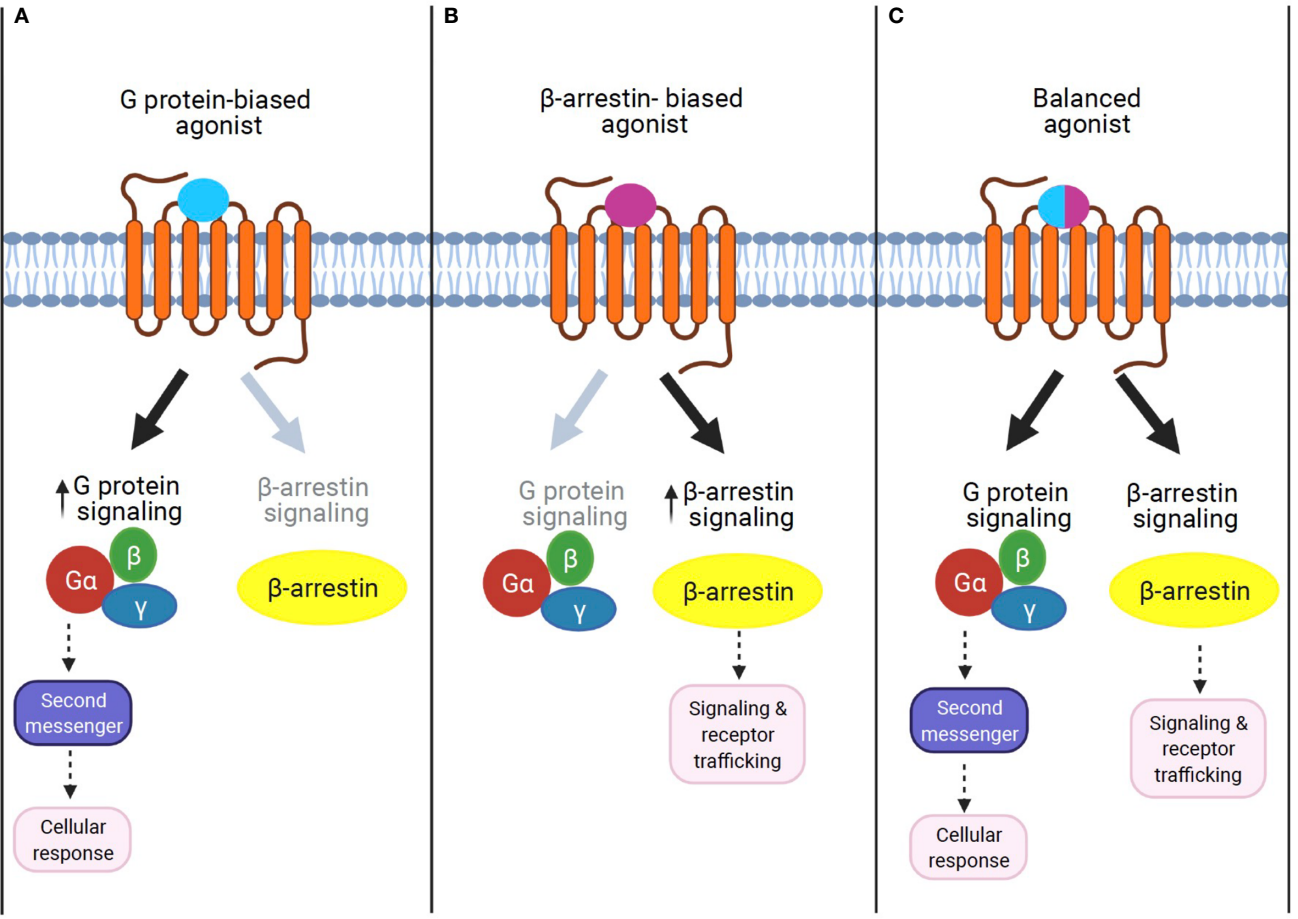
The concept of GPCR signaling: biased signaling. **(A)** G protein-biased agonist. Biased agonists selectively activate the GPCR-dependent signaling pathway. Previous studies demonstrate that sustained G protein-mediated signaling can affect cellular response through second messenger activation. **(B)** β-Arrestin biased agonist. Biased agonists selectively activate the β-arrestin-dependent signaling pathway. The β-arrestin-mediated signaling leads to distinct physiological outcomes. **(C)** Balanced agonist. Balanced agonists activate both the G protein- and β-arrestin-dependent signaling pathway.

G protein-coupled receptors (GPCRs) regulate virtually all metabolic processes, including glucose and energy homeostasis. In this review, we focus on GPCRs that function in metabolic disorders, particularly in T2D and obesity-related diseases. Several endogenous ligands such as free fatty acids and their receptors (e.g., GPR40, GPR41, GPR43, GPR84, GPR119, and GPR120) have been extensively studied in the regulation of insulin secretion, insulin sensitization, β-cell expansion, and glucose homeostasis (**Figure 1** and **Table 1**). Concomitantly, drugs that target these GPCRs in metabolic tissues have emerged as attractive T2D therapeutic targets as well (47). Thus, this review will discuss GPCRs and their signaling pathways (G protein-dependent and/or β-arrestin-dependent) that can be targeted pharmacologically to treat T2D by improving insulin sensitivity (**Figures 1** and **2**).

**Table 1:** Selection of GPCRs and their endogenous agonists act as signaling molecules.

GPCR	Endogenous ligands	Expression and metabolic effects	G-protein/β-arrestin signaling	Refs.
GPR35	KA; 2-acyl-LPA	Immune cells: anti-inflammatory	Gi; G12/13	(6–9)
		Enteroendocrine cells: ↑ CCK secretion		
		CNS: neuronal excitability and nociception		
		Adipocytes: ↑ lipolysis/energy expenditure		
GPR40	LCFAs	Endocrine pancreas: ↑ insulin secretion	Gq/11; β-arrestin2	(10–14)
		Enteroendocrine cells: ↑ GLP-1 and GIP secretion		
GPR41	SCFAs (acetate, propionate, butyrate)	Immune cells: anti-inflammatory	Gi/o; Gβγ; β-arrestin2	(15–20)
		Enteroendocrine cells: ↑ GLP-1 secretion		
		Endocrine pancreas: ↓ insulin secretion		
GPR43	SCFAs (acetate, propionate, butyrate)	Immune cells: anti-inflammatory	Gi/o; Gαq; β-arrestin2	(21–25)
		Adipocytes: ↓ lipolysis		
		Enteroendocrine cells: ↑ GLP-1 secretion		
		Endocrine pancreas: ↑ insulin secretion		
GPR81	Lactate	Adipocytes: ↓ lipolysis	Gi/o; β-arrestin2	(26–33)
		Ghrelin cells: ↓ Ghrelin secretion		
		Immune cells: anti-inflammatory		
GPR119	OEA; LPL; 2-MAG	Endocrine pancreas: ↑ insulin and glucagon; Enteroendocrine cells: ↑ GLP-1 and GIP secretion	Gs; β-arrestin2	(34–40)
GPR120	PUFAs (ω3-FAs; ω6-FAs)	Immune cells: anti-inflammatory	Gi/o; Gq/11 β-arrestin2	(41–46)
		Endocrine pancreas: ↓ SST secretion;		
		Stomach: ↓ ghrelin and SST secretion;		
		Adipocytes: ↑ insulin mediated glucose uptake		

KA, kynurenic acid; LPL, lysophosphatidic acid; LCFAs, long-chain fatty acids; SCFAs, short-chain fatty acids; OEA, oleylethanolamide; LPL, lysophospholipid; 2-MAG, 2-monoacyl-glycerol; PUFAs, polyunsaturated fatty acids; CCK, cholecystokinin; CNS, central nervous system; GLP-1, glucagon-like peptide 1; GIP, gastric inhibitory polypeptide; SST, somatostatin. ↑ indicates ‘increased’, ↓ indicates ‘decreased’.a

## GPCR Signaling Pathways in Metabolism

### G Protein-Dependent Mechanisms

GPCRs are the most common target of therapeutic drugs today. These seven transmembrane receptors (7TMRs) are synthesized, folded, and assembled in the endoplasmic reticulum, packed in vesicles, and transported to the plasma membrane (48). Upon binding to its cognitive ligands, GPCRs undergo a conformational change, which is transmitted to the cytoplasmic portion to couple with a heterotrimer (α, β, and γ subunits) of GTP-binding protein (G proteins) (49). GPCRs typically couple into a specific G protein such as G_s_, G_i_, G_q/11_, or G_12/13_. Coupling to G_s_ stimulates adenylate cyclase (AC) to increase cAMP levels, while coupling to G_i_ inhibits adenylate cyclase. G_q/11_ activation simulates phospholipase C (PLC) to hydrolyze membrane phospholipids to release inositol 1,4,5,-triphosphate (IP_3_) and diacylglycerols (DAGs), which then leads to increased intracellular calcium concentrations. G_q/11_ can also lead to PI3K and AKT activation (49–51). Following G protein activation, a family of G protein-coupled receptor kinases (GRKs) can phosphorylate the cytoplasmic domain of the GPCR, which recruits β-arrestin adapter molecule. Once recruited to the GPCR, β-arrestin can facilitate internalization of the receptor or propagate a separate signaling cascade mediating distinct biological effects.

### G Protein-Independent/β-Arrestin-Dependent Mechanisms

β-Arrestin1 (arrestin 2) and β-arrestin2 (arrestin 3) are ubiquitously expressed cytosolic adaptor proteins that were originally discovered for their inhibitory roles in GPCR signaling *via* the G protein-mediated signaling pathway (52). β-Arrestin1 and 2 share ~80% amino acid sequence identity and highly conserved structural features, but present unique, as well as shared roles in GPCR signaling and regulation (53). In response to receptor activation, β-arrestins can act as adaptor proteins to trigger the removal of activated GPCRs from the cell surface *via* clathrin-coated pits (54). This ability of β-arrestin1 and β-arrestin2 to terminate GPCR signaling through internalization is known as their classical or canonical actions (54, 55). While receptor desensitization requires β-arrestin interaction with activated GPCRs, β-arrestins can also transduce intracellular signaling as an adaptor protein (56, 57). For example, β-arrestins have been shown to form signaling scaffolds for mitogen-activated protein kinases (MAPKs) such as the extracellular signaling kinases (ERKs) and c-Jun N-terminal kinase 3 (JNK3) on endosomes with internalized GPCRs. Subsequently, β-arrestins have been known to promote some of these pathways even when the G protein activity is disabled. The finding that β-arrestins can mediate the G protein-independent signaling pathway of GPCRs led to the discovery that the two signaling pathways are pharmacologically distinct. In other words, it is possible to identify agonists that can selectively activate either G protein-dependent or β-arrestin-dependent signaling. Such agonists, which can selectively activate one or the other signaling pathway, are termed “biased agonists,” and this phenomenon of selective activation is termed “biased agonism” (58). Although “biased agonism” is often used in a sense to refer to “β-arrestin-biased agonism” in GPCR signaling (59, 60), it generally describes the disparity of the efficacies of agonists in activating signals mediated by different downstream effectors, for example, different G protein isoforms, G protein versus β-arrestin, or biases from many other signaling pathways (61). In some cases, the biased agonist could act as an antagonist or an inverse agonist for G protein-dependent signaling but as an agonist for β-arrestin-dependent signaling in a single GPCR (62, 63). Unlike G protein-mediated signaling pathways, which are transient and rapid, the β-arrestin-mediated pathway is often persistent and slow (58, 64, 65).

### GPCRs in Metabolic Tissues and Cells

A number of GPCRs have been extensively studied in metabolic tissues (i.e., white and brown adipose tissue, gut, liver, and pancreatic β-cells), which are shown to modulate metabolic response such as insulin secretion, glucose homeostasis, as well as energy expenditure and more (41, 47). Certain GPCRs play important roles in improving inflammation and insulin response in adipose tissue. Given their pleiotropic effects, GPCRs in white adipose tissue (WAT) and brown adipose tissue (BAT) are potential targets for the treatment of metabolic diseases (66–68). GPCRs are also involved in the regulation of insulin secretion. Although the molecular mechanisms of islet GPCR remain to be elucidated, functional studies of the β-cells have shown that activation of GPCRs can modulate β-cell signaling through alterations in intracellular levels of cAMP, IP_3_, and Ca^2+^, as well as in protein phosphorylation and acylation (23, 48, 69, 70). Such alterations modulate insulin secretion, indicating that β-cell GPCRs are promising targets for the development of antidiabetic therapeutics. Additionally, some GPCRs are abundantly expressed in macrophages and regulate diverse macrophage functions, including cell–cell contact, survival, chemotaxis, and the activation of inflammatory mediator production (42). These macrophage-enriched GPCRs are also implicated in metabolic dysfunction related to obesity. In fact, their agonists may interact with components of multiple pathways in macrophages to modulate signaling crosstalk with metabolic tissues, coordinating a precise and appropriate cellular response in order to improve the insulin signaling and other obesity-related metabolic disorders.

The following sections will discuss the role of GPCR signaling in key metabolic tissues (**Table 1**).

#### GPR35

GPR35 is a class A (rhodopsin-like) GPCR identified in 1998 (71). It is expressed in various tissues, such as central and peripheral nervous tissues, the gastrointestinal tract (GI), and lymphoid tissues (72–74). In the nervous tissues, several investigators have suggested that GPR35 activation regulates neuronal excitability, synaptic release (6), and nociception (73). In the GI tract, GPR35 has been linked to the development of gastric cancer (74), but it also actively modulates energy balance through the secretion of peptide hormones, such as cholecystokinin (CCK). GPR35 is coexpressed with the CCK1 receptor and the proton sensing receptors in GI vagal afferents neurons, suggesting that it may be part of the gut–brain signal axis that regulates energy balance (75).

The endogenous ligand for GPR35 has remained controversial. Kynurenic acid (KYNA), a tryptophan metabolite, was first proposed as a potential ligand candidate (72, 76). However, even at very high concentrations, KYNA seems to be almost inactive on human GPR35 (7). 2-Acyl lysophosphatidic acid is another endogenous ligand for GPR35. Oka et al. described that 2-acyl lysophosphatidic acid induces Ca^2+^ response, activates RhoA, increases the phosphorylation of ERKs, and also triggers the internalization of GPR35 in GPR35-expressing HEK293 cells (7). Additionally, the chemokine CXCL17 has also been proposed as a GPR35 endogenous agonist (8); however, subsequent studies by other teams (77, 78) have failed to support this study. Thus, despite the significant efforts to identify the real endogenous activator(s) for GPR35, GPR35 remains as a liganded orphan receptor.

Although the search for the selective and sensitive GPR35 ligands is underway, there are a number of studies to characterize the metabolic function of GPR35 in animal models. Agudelo et al. have shown that KYNA alleviates metabolic alterations triggered by high fat diet (HFD)-feeding, as it reduced weight gain, improved glucose tolerance, and remarkably reduced circulating TG levels. These effects of KYNA were associated with increased expression of adipose tissue thermogenic genes, specifically the expression of peroxisome proliferator-activated receptor-γ coactivator 1α (PGC-1α) in adipocytes. Interestingly, the effects of KYNA are lost in GPR35 KO mice, which are more susceptible to the effects of HFD-feeding, gaining more weight, developing glucose intolerance, and showing reduced browning of the subcutaneous adipose tissue (9). These findings identify a new metabolic role of GPR35 that can potentially be exploited for the treatment of obesity-related metabolic disorders and T2D.

#### GPR40

G protein‐coupled receptor 40 (GPR40), or free fatty acid receptor 1 (FFAR1), is found in pancreatic islets, being particularly enriched in the pancreatic β-cells (**Figure 1**). GPR40 can be activated by medium‐ to long‐chain free fatty acids (FFAs) (76, 79). Activation of GPR40 by FFAs or synthetic agonists enhances insulin secretion (10–12), partly through the amplification of intracellular calcium signaling in a glucose‐dependent manner (11, 12). GPR40 couples to the G_q/11_, leading to the formation of IP3 and increasing intracellular calcium. Although enhancement of glucose-stimulated insulin secretion (GSIS) in β-cells requires extracellular calcium signaling (79), GPR40 stimulation increases intracellular calcium, which is dependent on glucose levels (12) and is mediated through the activation of PLC and an L-type Ca^2+^ channel (80).

GPR40-mediated signal transduction is known to be primarily through G protein-dependent mechanisms (10, 12). However, GPR40 can also activate the functionally distinct G protein-independent, but β-arrestins-dependent signaling pathway (13). The GPR40–β-arrestin2-mediated signaling axis is functionally linked to insulin secretion (14). Further studies with various GPR40 agonists’ activation indicate that G protein- and β-arrestin-biased signaling can be differentially modulated by different ligands, thus eliciting ligand-specific responses (biased agonism; **Figure 2**). While GPR40 agonists, palmitic acid, and oleic acid act through G_q/11_-mediated mechanisms, the synthetic agonist TAK-875 can act as a β-arrestin2-biased agonist, engaging β-arrestin2-dependent signaling to induce the insulinotropic activity of GPR40 (13). The biased GPR40 activation has shown a promising potential as a therapeutic target to enhance insulin secretion in T2D (79), but phase III clinical trials with TAK-875 were recently terminated due to signs of liver toxicity in patients (81). Therefore, therapies based on GPR40 agonism provide an attractive alternative in the discovery of antidiabetic drugs, but further studies are needed to determine if potential side effects induced by this approach can be avoided.

#### GPR41

GPR41, also known as free fatty acid receptor 3 (FFA3), is expressed in adipose tissue, pancreas, spleen, lymph nodes, bone marrow, and peripheral blood mononuclear cells including monocytes (15–17). GPR41 and GPR43 (described in the next section) are activated by short-chain fatty acids (SCFAs) such as acetate, propionate, and butyrate, which are produced during dietary fiber fermentation by gut resident bacteria (76). Despite similarities in the receptor sequence, KO mice studies revealed contradictory results about the effects of GPR41 and/or GPR43 loss-of-function on metabolism (18, 19, 21–23, 69, 82). Those studies indicated opposite effects of the two SCFA sensing GPCRs on insulin secretion. GPR41 was found to inhibit glucose-dependent insulin secretion (69), while GPR43 was reported to potentiate insulin secretion (19, 23). Therefore, the effects of SCFAs on insulin secretion seem to be fine-tuned by the balance between GPR41 and GPR43 expression and activation. However, high selective agonists and antagonists for GPR41 and 43, as well as tissue-specific GPR41 and/or GPR43 KO mice, are required to fully elucidate the involvement of these receptors in SCFA-mediated effects. Since loss of GPR41 caused decreased GPR43 expression (83) and dual GPR41 and GPR43 KO mice exhibited higher insulin secretion and improved glucose tolerance, determination of specific function of each receptor in the regulation of insulin secretion is complex (84).

Both SCFA receptors, GPR41 and GPR43, seem to have a preference for G_i/o_ signaling, resulting in the inhibition of AC and the reduction in cAMP production (16, 19). In pancreatic islets, the activation of GPR41 by its endogenous ligand, propionate, inhibited the glucose-dependent insulin secretion through the Gα_i/o_ pathway (19). However, other G protein-dependent pathways may also be triggered by GPR41 activation, as it was observed that SCFAs and ketone bodies induced GPR41-mediated activation of sympathetic neurons through Gβγ-PLCβ-MAPK signaling, stimulating body energy expenditure and helping to maintain metabolic homeostasis (18). The role of β-arrestin signaling by GPR41 and GPR43 activation is not clear; however, in monocytes, it was described that GPR41 and GPR43 form a heteromer, which, in addition to enhancing Ca^2+^ signaling, also induced β-arrestin-2 recruitment (20).

Although promising, together these findings show how essential future studies are in order to more clearly define the role and mechanisms of GPR41 in insulin secretion, as well as the potential druggability of its agonists/antagonists to improve T2D and obesity-related metabolic abnormalities.

#### GPR43

G protein‐coupled receptor 43 (GPR43), also known as free fatty acid receptor 2 (FFA2), has been reported to be present in cells of the distal ileum, colon, and adipose tissue, with the highest expression found in immune cells such as monocytes and neutrophils (15, 16, 85). GPR43 appears to play a role during inflammation, as immune challenges such as LPS, TNFα, or granulocyte-macrophage colony stimulating factor (GM-CSF) were found to raise *GPR43* transcript levels in human monocytes (86, 87). Although GPR43 was not identified in the human adipose tissue (17), GPR43 inhibits lipolysis in mouse adipocytes (21). Adipocytes treated with GPR43 natural ligands, acetate and propionate, exhibit a reduction in lipolytic activity. Since this reduction in the lipolytic activity was abolished in adipocytes isolated from GPR43 KO mice, it seems to be the result of GPR43 activation (21). In addition to higher lipolysis and higher energy expenditure, deletion of GPR43 was also reported to improve the glucose homeostasis in obesity, as HFD-fed GPR43 KO mice exhibited lower body fat mass and increased insulin sensitivity (22). Despite the beneficial effects of the deletion of GPR43 in adipocytes, HFD-fed GPR43 KO mice exhibited dysfunctional β-cells, which showed reduced cell mass and lower expression of β-cell differentiation genes. Those abnormalities blunted the insulin secretion in GPR43 KO mice (23, 24). On the other hand, the treatment with acetate, endogenous GPR43 agonist, improved insulin secretion in mouse but not in human islets (23, 24).

It has been shown that acetate and synthetic GPR43 agonists can differently modulate GPR43 activation *via* coupling to multiple G protein pathways in mouse and human islets (69). PA ((S)-2-(4-chlorophenyl)-3,3-dimethyl-*N*-(5-phenylthiazol-2-yl)butanamide), a synthetic GPR43 agonist, potentiated insulin secretion in isolated murine islets, human islets, and Min6 cells *in vitro* by increasing intracellular IP3 and Ca^2+^ levels in a GPR43-, G_q_-, and PLC-dependent manner (23). However, another GPR43 synthetic agonist, 4-CMTB (4-Chloro-α-(1-methylethyl)-N-2-thiazolyl-benzeneacetamide), invariably inhibited GSIS in human pseudoislets, contrary to mouse islets, where it augmented GSIS (88). This finding that mouse and human islets responded differently to acetate and GPR43 agonists in GSIS assay will require close attention in future studies, since GPR43 is considered as a potential T2D target.

It has been proposed that β-arrestin2-mediated signaling can also be activated by GPR43 agonists, causing further inhibition of NF-κB and downregulation of its inflammatory gene targets (25). However, this is the only study where GPR43 and β-arrestin2 internalization was triggered by PA (25). Therefore, the idea of GPR43 biased agonism might be plausible, but more studies are necessary to verify its potential druggability for T2D and obesity-related metabolic disorders.

#### GPR81

GPR81 is a member of the hydroxyl-carboxylic acid receptor family. GPR81 is highly expressed in adipose tissue but also found in kidney, skeletal muscle, and liver (26, 89). Lactate is an endogenous ligand for GPR81. In adipocytes, the activation of GPR81 inhibits lipolysis by decreased cAMP and phosphorylation of PKA, which consequently reduces the activity of the hormone-sensitive lipase (27–29). GPR81 stimulated by lactate decreases intracellular cAMP and lipolysis, which was also found to work synergistically with insulin (90). In fact, Ahmed et al. observed that lactate and GPR81 unexpectedly functioned in an autocrine and paracrine loop to mediate insulin-induced antilipolytic effects (90). Additionally, GPR81 might also be linked to obesity, since HFD-fed GPR81 KO mice exhibited lower weight gain (90).

Obesity is also associated with increased inflammation, and several studies observed that GPR81 plays a role in inflammation. GPR81 expression in adipocytes and endothelial cells is reduced under inflammatory conditions (30, 31). Similarly, GPR81 expression is significantly decreased in the white adipose tissue of HFD-fed mice (91), as well as in adipocytes of *ob/ob* mice, an animal model of T2D characterized by high inflammation (30). Despite the clear link between inflammation and GPR81 function in the adipose tissue, the effects of GPR81 in macrophages and other immune cells are not fully understood. There are several reports that the expression of GPR81 in immune cells promote downregulation of the innate immune response (26, 32, 33). Hoque et al. observed that the immunosuppressive function of GPR81 was attributed to the downregulation of TLR- and/or NLRP3-mediated signaling (33). This GPR81-stimulated reduction of the inflammatory responses in macrophages/monocytes seems to be due to downstream signaling of GPR81 and β-arrestin2 and not due to a reduction in cAMP (G protein-dependent) (33). The exact mechanism for how GPR81 and β-arrestin2 counteract the NLRP3 and TLR pathways is currently unknown and will require further studies. Given the strong association between inflammation and obesity-related metabolic dysfunction, GPR81 biased agonism might be a valuable drug therapy for T2D and needs to be further investigated.

#### GPR119

GPR119 is a G_s_-coupled receptor that is expressed in pancreatic β-cells and gastrointestinal enteroendrocrine cells (34). GPR119 directly leads to an increase in insulin secretion in β-cells and promotes the release of both glucagon-like peptide-1 (GLP1) and gastric inhibitory polypeptide (GIP) in enteroendocrine cells (34–36). Thus, GPR119 stimulation can augment insulin secretion both by direct effects on β-cells and indirectly through GLP1 (34–36). GPR119 couples to G_s_ in response to several lipid-based agonists, with highly constitutive activity. GPR119 was originally described as a receptor for N-acylethanolamines, such as oleylethanolamide (OEA), which are generated locally in enterocytes (92). Lysophospholipids (LPL), another known class of endogenous GPR119 ligands, are directly absorbed from the diet or generated by catalysis of endogenous phospholipids (93). 2-Monoacylglycerols (2-MAGs), generated in high amounts during intestinal digestion of triglycerides, also bind and activate GPR119 (36). These findings illustrate that the most effective endogenous, fat-derived ligand for GPR119 is still ill defined, with OEA, LPL, or 2-monoacylglycerols being potential candidates (36, 76). Despite the debate about potential agonists, GPR119 signaling through G_s_ is well established, inducing cAMP accumulation and/or downstream activation of CREB (cAMP response element binding protein) in response to both natural ligands and synthetic GPR119 agonists (34, 37–39). GPR119 activation has also been proposed to induce ligand-mediated calcium release (94).

G protein-independent signaling *via* β-arrestin recruitment has only been sparsely described in the GPR119-mediated signaling pathway (40, 95). Hassing et al. described that GPR119 activation by OEA can trigger a β-arrestin biased signaling (40). Given the effects of GPR119 in β-cells (34–36), more studies are necessary to understand the effects of GPR119 biased agonism and investigate whether it might be plausible to stimulate the insulin release and treat T2D.

#### GPR120

GPR120 (*a.k.a.* free fatty acid receptor 4 (FFA4)) is the most abundant one among free fatty acid receptors in the mouse adipose tissue (17); however, GPR120 is also found in the pancreas, where expression is suggested to be restricted to the δ-cells (96), in lung (97), and in immune cells, specifically macrophages (41) (**Figure 1**). GPR120 is highly expressed in different types of macrophages (monocytes, Kupffer cells in the liver, osteoclasts in the bone, resident macrophages in the lung) and plays an important role in the regulation of inflammation (42). GPR120 is described as a receptor for omega-3 polyunsaturated fatty acids (ω3-FAs) (76), the activation of which reduces adipose tissue inflammation and protects against global insulin resistance (41, 43). The pathways coupled to GPR120 stimulation diverge between G protein- and β-arrestin-dependent pathways (98) and are critical for the regulation of metabolic or inflammatory processes (41). We and others have shown that GPR120 effectively responds to ω3-FAs, and the activation of GPR120 stimulates the PI3K/Akt pathway triggering GLUT4 translocation to the cell membrane and increasing glucose uptake in adipocytes by a G_q/11_-dependent mechanism, not by the β-arrestin-dependent pathway (41). In addition, Paschoal et al. showed that GPR120 agonist stimulation in adipocytes displayed biphasic ERK phosphorylation with G protein-mediated acute phase of ERK activation followed by a steady-state, β-arrestin-mediated ERK signaling pathway (44).

Although GPR120 stimulation leads to both G_q/11_ and β-arrestin-mediated pathway activation, it has been shown that the G protein-independent, β-arrestin2-dependent signaling pathway is responsible for the GPR120-mediated anti-inflammatory effects in macrophages (43). The activation of GPR120 signaling pathways regulate the macrophage phenotypic switch, influencing their response to inflammation and ability to migrate to other tissues (43).

Mechanistically, it has been shown that GPR120 activation and concomitant recruitment of β-arrestin-2 promote further interactions between β-arrestin-2 and TAB-1. This receptor mediated β-arrestin-2/TAB-1 interaction is suggested to prevent the formation of a TAB-1/TAK-1 (transforming growth factor kinase) complex, blocking the subsequent signaling that results in the activation of inflammatory responses (41, 43). β-Arrestin-2 pull-down experiments demonstrated the physical interaction between β-arrestin-2, GPR120, and TAB-1 in macrophages after stimulation with DHA or DHA plus LPS, respectively (41). Similar results have been reported using a synthetic agonist of GPR120 to replace the fatty acid: compound A (cpdA) (41). This molecule produced anti-inflammatory effects in macrophages in both *in vitro* and *in vivo* models (43). Treatment of primary macrophages with cpdA in conjunction with LPS inhibited the phosphorylation of many of the previously discussed phospho-regulated kinases that are typically activated in inflammatory processes (e.g., p-IKK, p-JNK, pTAK-1) (43).

The functions of GPR120 in the adipose tissue can be linked to pathological ramifications of obesity (41, 43, 99, 100); thus, GPR120 as a target for the development of novel compounds to treat metabolic syndrome becomes a very promising approach. However, efforts to identify or generate GPR120 biased agonists are still ongoing (101).

#### GLP-1R

The glucagon-like peptide-1 receptor (GLP-1R) is a GPCR predominantly expressed in the β-cells, intestine, heart, breast, and brain (102). GLP-1R mediates a number of physiological effects, and due to its functions, the GLP-1R is a major therapeutic target for treatment of type 2 diabetes and obesity. It increases insulin secretion by direct stimulation of gene expression, synthesis, and secretion of insulin from pancreatic β-cells; it enhances β-cell mass by increasing neogenesis and proliferation, while decreasing apoptosis; it suppresses glucagon secretion and inhibits gastric emptying; and extra-pancreatically GLP-1 also acts at a range of sites such as the nervous system, where it signals satiety, reducing food intake (103–105). A large number of GLP1-based therapeutics are already well established, and the scientific basis underlying these therapeutic approaches is quite advanced; therefore, this subject will only be briefly reviewed.

The GLP-1R physiological effects rely on downstream signaling pathways mediated by GLP-1 interaction. This receptor activates Gαs proteins enhancing the formation of cAMP; however, it can pleiotropically interact with multiple other G proteins including Gi/o and Gq proteins, leading to the activation of downstream signaling pathways that include the mobilization of intracellular calcium and the phosphorylation of mitogen activated protein kinases such as extracellular regulated kinases 1/2 (ERK1/2), protein kinase B (Akt/PKB), phosphoinositide 3 (PI3) kinase, and p38, among others (102, 106). In addition to signaling *via* G proteins, the GLP-1R can also promote β-arrestin-1 biased, non-G protein-mediated cellular signaling (47, 102).

Several groups have reported GLP-1R biased agonism; for instance, the drugs Oxyntomodulin and exendin are GLP-1R biased agonists that recruit β-arrestin in a higher potency than G protein-mediated cAMP and ERK1/2 activation (102, 107, 108). In addition to direct evidence of biased agonism by different peptide ligands, there is also some evidence that the kinetics of GLP-1R internalization and recycling mediated by distinct peptides may contribute to biased agonism profiles. The potencies of GLP-1 and exendin-induced internalization are 10-fold higher than that of liraglutide (107). With emerging evidence that internalized GLP-1Rs can continue to signal inside the cell and that spatial-temporal control of signaling pathways promotes distinct physiological functions, this ability of different ligands to promote different kinetics of internalization and recycling of the GLP-1R may contribute to the observed ligand-biased agonism (107). Taken together, all these studies indicate that biased signaling occurs at this receptor, and this may have the potential to be exploited in drug development.

Together, these findings highlight the current lack in understanding the role of the biased G protein- and/or β-arrestin-mediated mechanism in GPCR signaling, even for well-known GPCRs. Therefore, future studies that identify and synthesize biased agonists for each GPCR, as well as explore the biased signaling mechanism in target tissues, are imperative (**Figure 2**). In the next section, we will discuss promising approaches that β-arrestin biased activation may uniquely represent to treat T2D and obesity-related comorbidities.

## Role of β-Arrestins in Metabolism

Among four members of the arrestin family, β-arrestin1 (arrestin 2) and β-arrestin2 (arrestin-3) are widely expressed in different tissues and implicated in many GPCR signaling pathways to regulate cellular responses. β-Arrestin signaling serves multiple purposes; it can modulate the activity of several cellular signaling proteins such as PI3K and AKT (109), c-Src, MAPKs, cAMP phosphodiesterase, calmodulin, protein phosphatases, ubiquitin ligases, deubiquitinating enzymes, and many others (54, 110). It is still unclear whether these noncanonical β-arrestin functions require prior recruitment by GPCRs, as the main function of these proteins is to terminate GPCR cellular pathways. Since β-arrestins can act independently as signaling molecules, therefore, it is important to understand their independent role in metabolic homeostasis (111). An increasing number of studies have focused on the role of β-arrestins in metabolic tissues, such as white and brown adipose tissue, liver, and the pancreatic β cell. **Table 2** and the following sections will provide new insights for the role of β-arrestins in metabolic tissues, regardless of G protein-dependent or independent signaling.

**Table 2:** Metabolic functions of β-arrestins in distinct cell types and associated GPCRs.

*Cell type*s	*Mouse* models	*Metabolic phenotypes*	*Molecular mechanism*s	*Associat*e*d* GPCRs	*Ref*s.
Adipocytes	βarr1-AKO	Glucose intolerance and insulin resistance (on HFD)	↑Myostain expression in BAT	β3-AR; GPR35; GPR43; GPR120	(112, 113)
	βarr1-AOE	Improved glucose tolerance and insulin sensitivity (on HFD)	↓Myostain expression in BAT		
	βarr2-AKO	Adiposity↓ (on HFD) and improved glucose tolerance; ↓ HFD-induced metabolic disorders	Enhanced β3-AR signaling		
Hepatocytes	βarr2-HKO	Impaired glucose tolerance; Reduced HFD-induced metabolic deficits	Enhanced GCGR signaling; Inhibited GCGR signaling	GCGR	(114)
	βarr2-HOE				
β-Cells	βarr1-βKO	Decreased the efficiency of SU drugs in insulin secretion	Damaged EPAC2 function	GPR40; GPR41; GPR43; GPR119	(115, 116)
	βarr2-βKO	Impaired glucose tolerance and insulin secretion (on HFD)	CAMKII inactivated function		
	βarr2-βOE	Improved glucose tolerance and insulin sensitivity (on HFD)	Enhanced CAMKII activity		
Skeletal muscle cells	βarr2-SMKO	Mild improved glucose tolerance and insulin sensitivity (on HFD)	↑Insulin-induced AKT activation in SKM	GPR?	(117)
AgRP neurons	βarr1-AgKO βarr1-AgOE	Glucose intolerance and insulin resistance (on HFD)	βarr1 deficiency prevents insulin from AgRP neurons	GPR35	(112)
		Improved glucose tolerance and insulin sensitivity (on HFD)	Increased insulin sensitivity in AgRP neurons		

KO, knockout; OE, overexpression; βarr, β-arrestin; HFD, high fat diet; BAT, brown adipose tissue; β3-AR, β3 adrenergic receptor; GCGR, glucagon receptor; EPAC2, exchange protein directly activated by cAMP 2,; CAMKII, Ca^2+^/calmodulin-dependent protein kinase II; AKT, protein kinase B; SKM, skeletal muscle; AgRP, Agouti-related protein. ↑ indicates ‘increased’, ↓ indicates ‘decreased’.

### Adipocytes

It is known that white adipocytes play essential roles in storing extra lipids as energy and releasing fatty acids as resources. In contrast, mutilocular brown adipocytes oxidize fatty acids and other substrates to produce heat for maintaining body temperature in mammals. During the past few years, many laboratories have used mouse genetics to identify the role of β-arrestin1 and 2 in adipocyte function and whole-body glucose homeostasis (118, 119). Pydi et al. (120) reported that selectively lacked β-arrestin1 in adipocyte (β-arr1 AKO) mice on HFD are glucose intolerance and insulin resistance (**Table 2**). On the other hand, mice overexpressing β-arrestin1 in adipocytes (β-arr1 AOE) were protected against HFD-induced metabolic deficits (120). In contrast to β-arrestin1, mice lacking β-arrestin2 in adipocytes (βarr2-AKO) display improved metabolic phenotypes (118). These findings convincingly identify that β-arrestin1 and 2 not only are required for the maintenance of glucose homeostasis in their own right but also strongly suggest that strategies aiming to enhance β-arrestin activity in adipocytes may be beneficial for the treatment of T2D and obesity-related metabolic disorders.

### β-Cells

Insulin resistance is a key etiological factor in the development of T2D. It can be triggered by desensitization of insulin signaling at several steps (2). Studies have demonstrated that β-arrestin1 can function as a nodal point for heterologous desensitization and crosstalk between receptor tyrosine kinases (RTKs) and GPCR signaling pathways (121, 122). β-Arrestin1 has been showed to modulate ubiquitination and degradation of one major substrate in the insulin cascade, the RTK, and insulin receptor substrate (IRS) (122). β-Arrestin1 competes with IRS proteins for ubiquitination and degradation, such that β-arrestin1 deficiency accelerates insulin-induced IRS degradation, exacerbating cellular insulin resistance, whereas overexpression of β-arrestin1 restrains this process, leading to increased insulin signaling downstream of IRS-1 and improving cellular insulin sensitivity (122).

Pancreatic β-cell specific β-arrestin2 KO mice on HFD (βarr2-βKO mice) showed impaired insulin release and glucose tolerance, whereas the β-cell specific overexpression of β-arrestin2 mice on HFD (βarr2-βOE mice) exhibited improved GSIS and glucose tolerance compared to HFD-fed control mice (116). These data indicate that β-arrestin2 act as an important regulator of β-cell function.

Impaired β-cell function is a major etiological defect underlying T2D. The failure to maintain compensatory hyperinsulinemia and the following decrease in plasma insulin levels is a key cause of hyperglycemia (2, 4, 5). Therapeutic measures to increase endogenous insulin secretion, and, indeed, administration of exogenous insulin itself, have been the cornerstones of T2D treatment for decades. In recent years, several developments have emerged that focus attention on the role of β-arrestins as therapeutic targets to enhance β-cell function and lower glucose levels.

### Agouti-Related Peptide (AgRP) Neurons

Different areas of brain play key roles in regulating and maintaining euglycemia. Neuronal subpopulations of the arcuate nucleus (ARC) of the hypothalamus, which synthesize and release agouti-related peptide (AgRP), have been studied extensively in relation to metabolic function (123). Numerous studies have shown that AgRP neurons play a key role in regulating food intake and energy homeostasis (124–127). Recent studies have shown that mice lacking β-arrestin1 in AgRP neurons on HFD displayed impaired glucose tolerance and insulin sensitivity accompanied with liver steatosis and increase in the plasma FFA level (112). Interestingly, they found that mice with specific deletion of β-arrestin1 in AgRP neurons (βarr1-AgKO) have increased PKA activity in adipose tissue, resulting in the accumulated lipolysis (112). In contrast, a mouse model where β-arrestin1 was overexpressed in these neurons (βarr1-AgOE) has significantly improved glucose and insulin tolerance (112). Collectively, these metabolic phenotypes may provide a novel way to improve glucose tolerance and insulin sensitivity in the AgRP neurons through the increased activation of β-arrestin1. Further studies are necessary to delineate the importance of GPCR signaling and the association between β-arrestin1 and related GPCRs in AgRP neurons.

### Hepatocytes

Hepatocytes play a significant role in T2D by controlling lipid metabolism and whole body glucose homeostasis (57). Insulin and glucagon are the two major hormones that regulate the metabolic function of hepatocytes (128). Many studies focus on the G_s_-coupled glucagon receptor (GCGR) in hepatocytes. The GCGR can trigger the cAMP/PKA-dependent signaling pathway to promote gluconeogenesis and glycogen breakdown, thereby increasing hepatic glucose production (129, 130).

Zhu et al. generated liver (hepatocyte) specific β-arrestin1 and β-arrestin2 KO mice (βarr1-HKO and βarr2-HKO) (131). While βarr1-HKO mice did not show any significant difference in metabolic phenotype compared to the control mice, βarr2-HKO mice displayed impaired glucose tolerance and hyperglycemia (131). When βarr2-HKO mice were treated with anti-GCGR antibody, the blood glucose level of these mice was back to the normal level. However, in mice where hepatocytes overexpressed β-arrestin2 (βarr2-HOE), the opposite metabolic phenotype was observed (131). Consistent with *in vivo* studies, they also found that glucagon treatment in primary hepatocytes isolated from control mice caused the internalization of the GCGR, whereas this effect was absent in hepatocytes isolated from βarr2-HKO mice (131). Collectively, both *in vivo* and *in vitro* data demonstrate that β-arrestin2 plays a negative role in GCGR signaling.

### Skeletal Muscle

Skeletal muscle is a major insulin target tissue for regulating whole body glucose homeostasis. Skeletal muscle specific β-arrestin2 KO mice (βarr2-SMKO) on HFD displayed slight improvements of glucose tolerance and insulin sensitivity (117). More detailed studies need to be done to illustrate how β-arrestin2 deficiency in skeletal muscle influences insulin signaling. In summary, skeletal muscle expressed GPCRs represent promising therapeutic targets for modulating insulin sensitivity and treating T2D and obesity-related metabolic disorders.

## Concluding Remarks and Perspectives

GPCRs are uniquely druggable targets that form the basis of the leading antidiabetic treatments, as evidenced by the large number of GLP1R/GLP1 therapies currently in use. Additionally, GPCRs that can influence insulin resistance, β-cell dysfunction, or both have recently been identified, and preclinical studies hold great promise. In addition, β-arrestins are crucial regulators of GPCR signaling. Although aspects of the GPCR/β-arrestin signaling network had been previously well established, the novelty of the recent studies highlighted in this review is the ability of β-arrestins to orchestrate a complex signaling network with or without GPCR activation that specifically controls metabolic homeostasis. Studies with β-arrestin KO mice have provided several key insights into the physiological implications of β-arrestin-dependent signaling. The multitudes of important metabolic processes that are regulated by β-arrestins offer new perspectives for the development of novel classes of therapeutic agents for the treatment of T2D and obesity-related pathophysiological conditions. Biased agonists for several GPCRs present a unique opportunity to explore the possibilities of developing a novel class of drugs. Such drugs may include G protein- or β-arrestin biased agonists that can interrupt or enhance metabolically relevant interactions of β-arrestin1 and β-arrestin2 with key signaling molecules. In this regard, additional preclinical studies are warranted to further analyze the effect of potential G protein- or β-arrestin biased signaling machinery to instruct novel therapeutic regimes for the treatment of metabolic disease.

## Author Contributions

All authors listed have made a substantial, direct, and intellectual contribution to the work and approved it for publication.

## Funding

This work was supported by grant from the NIH (R01-DK108773 to DO) and the American Heart Association (14SDG19880020 to DO).

## Conflict of Interest

The authors declare that the research was conducted in the absence of any commercial or financial relationships that could be construed as a potential conflict of interest.

## Publisher’s Note

All claims expressed in this article are solely those of the authors and do not necessarily represent those of their affiliated organizations, or those of the publisher, the editors and the reviewers. Any product that may be evaluated in this article, or claim that may be made by its manufacturer, is not guaranteed or endorsed by the publisher.

## References

[1] Control CfD, Prevention. National Diabetes Statistics Report, 2020. Atlanta, GA: Centers for Disease Control and Prevention, US Department of Health and Human Services (2020) p. 12–5.

[2] Kahn SE Hull RL Utzschneider KM. Mechanisms Linking Obesity to Insulin Resistance and Type 2 Diabetes. Nature (2006) 444(7121):840–6. 10.1038/nature05482 17167471

[3] Barnes AS. The Epidemic of Obesity and Diabetes: Trends and Treatments. Texas Heart Institute J (2011) 38(2):142–4. PMC3066828 21494521

[4] Cerf ME. Beta Cell Dysfunction and Insulin Resistance. Front Endocrinol (2013) 4:37. 10.3389/fendo.2013.00037 PMC3608918 23542897

[5] Prentki M Nolan CJ. Islet Beta Cell Failure in Type 2 Diabetes. J Clin Invest (2006) 116(7):1802–12. 10.1172/JCI29103 PMC1483155 16823478

[6] Guo J Williams DJ Puhl HL Ikeda SR. Inhibition of N-Type Calcium Channels by Activation of GPR35, an Orphan Receptor, Heterologously Expressed in Rat Sympathetic Neurons. J Pharmacol Exp Ther (2008) 324(1):342–51. 10.1124/jpet.107.127266 17940199

[7] Oka S Ota R Shima M Yamashita A Sugiura T. GPR35 Is a Novel Lysophosphatidic Acid Receptor. Biochem Biophys Res Commun (2010) 395(2):232–7. 10.1016/j.bbrc.2010.03.169 20361937

[8] Maravillas-Montero JL Burkhardt AM Hevezi PA Carnevale CD Smit MJ Zlotnik A. Cutting Edge: GPR35/CXCR8 I the Receptor of the Mucosal Chemokine CXCL17. J Immunol (Baltimore Md: 1950) (2015) 194(1):29–33. 10.4049/jimmunol.1401704 PMC4355404 25411203

[9] Agudelo LZ Ferreira DMS Cervenka I Bryzgalova G Dadvar S Jannig PR. Kynurenic Acid and Gpr35 Regulate Adipose Tissue Energy Homeostasis and Inflammation. Cell Metab (2018) 27(2):378–92.e5. 10.1016/j.cmet.2018.01.004 29414686

[10] Tan CP Feng Y Zhou Y-P Eiermann GJ Petrov A Zhou C. Selective Small-Molecule Agonists of G Protein–Coupled Receptor 40 Promote Glucose-Dependent Insulin Secretion and Reduce Blood Glucose in Mice. Diabetes (2008) 57(8):2211–9. 10.2337/db08-0130 PMC2494688 18477808

[11] Lin DC-H Zhang J Zhuang R Li F Nguyen K Chen M. AMG 837: A Novel GPR40/FFA1 Agonist That Enhances Insulin Secretion and Lowers Glucose Levels in Rodents. PloS One (2011) 6(11):e27270. 10.1371/journal.pone.0027270 22087278 PMC3210765

[12] Fujiwara K Maekawa F Yada T. Oleic Acid Interacts With GPR40 to Induce Ca2+ Signaling in Rat Islet β-Cells: Mediation by PLC and L-Type Ca2+ Channel and Link to Insulin Release. Am J Physiol Endocrinol Metab (2005) 289(4):E670–7. 10.1152/ajpendo.00035.2005 15914509

[13] Mancini AD Bertrand G Vivot K Carpentier É Tremblay C Ghislain J. β-Arrestin Recruitment and Biased Agonism at Free Fatty Acid Receptor 1. J Biol Chem (2015) 290(34):21131–40. 10.1074/jbc.M115.644450 PMC4543669 26157145

[14] Qian J Wu C Chen X Li X Ying G Jin L. Differential Requirements of Arrestin-3 and Clathrin for Ligand-Dependent and -Independent Internalization of Human G Protein-Coupled Receptor 40. Cell Signal (2014) 26(11):2412–23. 10.1016/j.cellsig.2014.07.019 25038452

[15] Brown AJ Goldsworthy SM Barnes AA Eilert MM Tcheang L Daniels D. The Orphan G Protein-Coupled Receptors GPR41 and GPR43 Are Activated by Propionate and Other Short Chain Carboxylic Acids. J Biol Chem (2003) 278(13):11312–9. 10.1074/jbc.M211609200 12496283

[16] Le Poul E Loison C Struyf S Springael J-Y Lannoy V Decobecq M-E. Functional Characterization of Human Receptors for Short Chain Fatty Acids and Their Role in Polymorphonuclear Cell Activation. J Biol Chem (2003) 278(28):25481–9. 10.1074/jbc.M301403200 12711604

[17] Amisten S Neville M Hawkes R Persaud SJ Karpe F Salehi A. An Atlas of G-Protein Coupled Receptor Expression and Function in Human Subcutaneous Adipose Tissue. Pharmacol Ther (2015) 146:61–93. 10.1016/j.pharmthera.2014.09.007 25242198

[18] Kimura I Inoue D Maeda T Hara T Ichimura A Miyauchi S. Short-Chain Fatty Acids and Ketones Directly Regulate Sympathetic Nervous System *via* G Protein-Coupled Receptor 41 (GPR41). Proc Natl Acad Sci (2011) 108(19):8030–5. 10.1073/pnas.1016088108 PMC3093469 21518883

[19] Priyadarshini M Layden BT. FFAR3 Modulates Insulin Secretion and Global Gene Expression in Mouse Islets. Islets (2015) 7(2):e1045182. 10.1080/19382014.2015.1045182 26091414 PMC4878265

[20] Ang Z Xiong D Wu M Ding JL. FFAR2-FFAR3 Receptor Heteromerization Modulates Short-Chain Fatty Acid Sensing. FASEB J: Off Publ Fed Am Societies Exp Biol (2018) 32(1):289–303. 10.1096/fj.201700252RR PMC5731126 28883043

[21] Ge H Li X Weiszmann J Wang P Baribault H Chen J-L. Activation of G Protein-Coupled Receptor 43 in Adipocytes Leads to Inhibition of Lipolysis and Suppression of Plasma Free Fatty Acids. Endocrinology (2008) 149(9):4519–26. 10.1210/en.2008-0059 18499755

[22] Bjursell M Admyre T Göransson M Marley AE Smith DM Oscarsson J. Improved Glucose Control and Reduced Body Fat Mass in Free Fatty Acid Receptor 2-Deficient Mice Fed a High-Fat Diet. Am J Physiol Endocrinol Metab (2011) 300(1):E211–20. 10.1152/ajpendo.00229.2010 20959533

[23] McNelis JC Lee YS Mayoral R van der Kant R Johnson AM Wollam J. GPR43 Potentiates β-Cell Function in Obesity. Diabetes (2015) 64(9):3203–17. 10.2337/db14-1938 PMC4542437 26023106

[24] Villa SR Priyadarshini M Fuller MH Bhardwaj T Brodsky MR Angueira AR. Loss of Free Fatty Acid Receptor 2 Leads to Impaired Islet Mass and Beta Cell Survival. Sci Rep (2016) 6:28159. 10.1038/srep28159 27324831 PMC4914960

[25] Lee SU In HJ Kwon MS Park BO Jo M Kim MO. β-Arrestin 2 Mediates G Protein-Coupled Receptor 43 Signals to Nuclear Factor-κb. Biol Pharm Bull (2013) 36(11):1754–9. 10.1248/bpb.b13-00312 23985900

[26] Brown TP Ganapathy V. Lactate/GPR81 Signaling and Proton Motive Force in Cancer: Role in Angiogenesis, Immune Escape, Nutrition, and Warburg Phenomenon. Pharmacol Ther (2020) 206:107451. 10.1016/j.pharmthera.2019.107451 31836453

[27] Cai T-Q Ren N Jin L Cheng K Kash S Chen R. Role of GPR81 in Lactate-Mediated Reduction of Adipose Lipolysis. Biochem Biophys Res Commun (2008) 377(3):987–91. 10.1016/j.bbrc.2008.10.088 18952058

[28] Ge H Weiszmann J Reagan JD Gupte J Baribault H Gyuris T. Elucidation of Signaling and Functional Activities of an Orphan GPCR, Gpr81. J Lipid Res (2008) 49(4):797–803. 10.1194/jlr.M700513-JLR200 18174606

[29] Liu C Wu J Zhu J Kuei C Yu J Shelton J. Lactate Inhibits Lipolysis in Fat Cells Through Activation of an Orphan G-Protein-Coupled Receptor, GPR81. J Biol Chem (2009) 284(5):2811–22. 10.1074/jbc.M806409200 19047060

[30] Feingold KR Moser A Shigenaga JK Grunfeld C. Inflammation Inhibits GPR81 Expression in Adipose Tissue. Inflammation Research: Off J Eur Histamine Res Soc [et al] (2011) 60(10):991–5. 10.1007/s00011-011-0361-2 21751047

[31] Boitsova EB Morgun AV Osipova ED Pozhilenkova EA Martinova GP Frolova OV. The Inhibitory Effect of LPS on the Expression of GPR81 Lactate Receptor in Blood-Brain Barrier Model In Vitro. J Neuroinflamm (2018) 15(1):196. 10.1186/s12974-018-1233-2 PMC6030740 29973231

[32] Ranganathan P Shanmugam A Swafford D Suryawanshi A Bhattacharjee P Hussein MS. GPR81, a Cell-Surface Receptor for Lactate, Regulates Intestinal Homeostasis and Protects Mice From Experimental Colitis. J Immunol (Baltimore Md: 1950) (2018) 200(5):1781–9. 10.4049/jimmunol.1700604 PMC5858928 29386257

[33] Hoque R Farooq A Ghani A Gorelick F Mehal WZ. Lactate Reduces Liver and Pancreatic Injury in Toll-Like Receptor- and Inflammasome-Mediated Inflammation *via* GPR81-Mediated Suppression of Innate Immunity. Gastroenterology (2014) 146(7):1763–74. 10.1053/j.gastro.2014.03.014 PMC4104305 24657625

[34] Chu ZL Carroll C Alfonso J Gutierrez V He H Lucman A. A Role for Intestinal Endocrine Cell-Expressed G Protein-Coupled Receptor 119 in Glycemic Control by Enhancing Glucagon-Like Peptide-1 and Glucose-Dependent Insulinotropic Peptide Release. Endocrinology (2008) 149(5):2038–47. 10.1210/en.2007-0966 18202141

[35] Lauffer L Iakoubov R Brubaker PL. GPR119: “Double-Dipping” for Better Glycemic Control. Endocrinology (2008) 149(5):2035–7. 10.1210/en.2008-0182 18427153

[36] Hansen HS Rosenkilde MM Holst JJ Schwartz TW. GPR119 as a Fat Sensor. Trends Pharmacol Sci (2012) 33(7):374–81. 10.1016/j.tips.2012.03.014 22560300

[37] Hansen KB Rosenkilde MM Knop FK Wellner N Diep TA Rehfeld JF. 2-Oleoyl Glycerol Is a GPR119 Agonist and Signals GLP-1 Release in Humans. J Clin Endocrinol Metab (2011) 96(9):E1409–17. 10.1210/jc.2011-0647 21778222

[38] Yoshida S Ohishi T Matsui T Tanaka H Oshima H Yonetoku Y. Novel GPR119 Agonist AS1535907 Contributes to First-Phase Insulin Secretion in Rat Perfused Pancreas and Diabetic Db/Db Mice. Biochem Biophys Res Commun (2010) 402(2):280–5. 10.1016/j.bbrc.2010.10.015 20937249

[39] Ning Y O’neill K Lan H Pang L Shan L Hawes B. Endogenous and Synthetic Agonists of GPR119 Differ in Signalling Pathways and Their Effects on Insulin Secretion in MIN6c4 Insulinoma Cells. Br J Pharmacol (2008) 155(7):1056–65. 10.1038/bjp.2008.337 PMC2528830 18724386

[40] Hassing HA Fares S Larsen O Pad H Hauge M Jones RM. Biased Signaling of Lipids and Allosteric Actions of Synthetic Molecules for GPR119. Biochem Pharmacol (2016) 119:66–75. 10.1016/j.bcp.2016.08.018 27569424

[41] Oh DY Talukdar S Bae EJ Imamura T Morinaga H Fan W. GPR120 Is an Omega-3 Fatty Acid Receptor Mediating Potent Anti-Inflammatory and Insulin-Sensitizing Effects. Cell (2010) 142(5):687–98. 10.1016/j.cell.2010.07.041 PMC2956412 20813258

[42] Lattin J Zidar DA Schroder K Kellie S Hume DA Sweet MJ. G-Protein-Coupled Receptor Expression, Function, and Signaling in Macrophages. J Leukocyte Biol (2007) 82(1):16–32. 10.1189/jlb.0107051 17456803

[43] Oh DY Walenta E Akiyama TE Lagakos WS Lackey D Pessentheiner AR. A Gpr120-Selective Agonist Improves Insulin Resistance and Chronic Inflammation in Obese Mice. Nat Med (2014) 20(8):942–7. 10.1038/nm.3614 PMC4126875 24997608

[44] Paschoal VA Walenta E Talukdar S Pessentheiner AR Osborn O Hah N. Positive Reinforcing Mechanisms Between GPR120 and Pparγ Modulate Insulin Sensitivity. Cell Metab (2020) 31(6):1173–88.e5. 10.1016/j.cmet.2020.04.020 32413335 PMC7337476

[45] Ahn S Shenoy SK Wei H Lefkowitz RJ. Differential Kinetic and Spatial Patterns of β-Arrestin and G Protein-Mediated ERK Activation by the Angiotensin II Receptor*. J Biol Chem (2004) 279(34):35518–25. 10.1074/jbc.M405878200 15205453

[46] Tohgo A Pierce KL Choy EW Lefkowitz RJ Luttrell LM. β-Arrestin Scaffolding of the ERK Cascade Enhances Cytosolic ERK Activity But Inhibits ERK-Mediated Transcription Following Angiotensin AT1a Receptor Stimulation*. J Biol Chem (2002) 277(11):9429–36. 10.1074/jbc.M106457200 11777902

[47] Oh DY Olefsky JM. G Protein-Coupled Receptors as Targets for Anti-Diabetic Therapeutics. Nat Rev Drug Discov (2016) 15(3):161–72. 10.1038/nrd.2015.4 26822831

[48] Winzell MS Ahrén B. G-Protein-Coupled Receptors and Islet Function—Implications for Treatment of Type 2 Diabetes. Pharmacol Ther (2007) 116(3):437–48. 10.1016/j.pharmthera.2007.08.002 17900700

[49] Kobilka BK. G Protein Coupled Receptor Structure and Activation. Biochim Biophys Acta (2007) 1768(4):794–807. 10.1016/j.bbamem.2006.10.021 17188232 PMC1876727

[50] Gether U. Uncovering Molecular Mechanisms Involved in Activation of G Protein-Coupled Receptors. Endocrine Rev (2000) 21(1):90–113. 10.1210/edrv.21.1.0390 10696571

[51] Katritch V Cherezov V Stevens RC. Structure-Function of the G Protein-Coupled Receptor Superfamily. Annu Rev Pharmacol Toxicol (2013) 53:531–56. 10.1146/annurev-pharmtox-032112-135923 PMC3540149 23140243

[52] Jean-Charles PY Kaur S Shenoy SK. G Protein-Coupled Receptor Signaling Through β-Arrestin-Dependent Mechanisms. J Cardiovasc Pharmacol (2017) 70(3):142–58. 10.1097/FJC.0000000000000482 PMC5591062 28328745

[53] Ranjan R Dwivedi H Baidya M Kumar M Shukla AK. Novel Structural Insights Into GPCR–β-Arrestin Interaction and Signaling. Trends Cell Biol (2017) 27(11):851–62. 10.1016/j.tcb.2017.05.008 28651823

[54] Pydi SP Barella LF Meister J Wess J. Key Metabolic Functions of β-Arrestins: Studies With Novel Mouse Models. Trends Endocrinol Metab (2020). (2021) 32(2):118–29. 10.1016/j.tem.2020.11.008 PMC7855863 33358450

[55] Pierce KL Lefkowitz RJ. Classical and New Roles of β-Arrestins in the Regulation of G-PROTEIN-COUPLED Receptors. Nat Rev Neurosci (2001) 2(10):727–33. 10.1038/35094577 11584310

[56] Oakley RH Laporte SA Holt JA Barak LS Caron MG. Association of Beta-Arrestin With G Protein-Coupled Receptors During Clathrin-Mediated Endocytosis Dictates the Profile of Receptor Resensitization. J Biol Chem (1999) 274(45):32248–57. 10.1074/jbc.274.45.32248 10542263

[57] Pydi SP Barella LF Meister J Wess J. Key Metabolic Functions of β-Arrestins: Studies With Novel Mouse Models. Trends Endocrinol Metab (2021) 32(2):118–29. 10.1016/j.tem.2020.11.008 PMC7855863 33358450

[58] Shukla AK Xiao K Lefkowitz RJ. Emerging Paradigms of Beta-Arrestin-Dependent Seven Transmembrane Receptor Signaling. Trends Biochem Sci (2011) 36(9):457–69. 10.1016/j.tibs.2011.06.003 PMC3168679 21764321

[59] Drake MT Violin JD Whalen EJ Wisler JW Shenoy SK Lefkowitz RJ. Beta-Arrestin-Biased Agonism at the Beta2-Adrenergic Receptor. J Biol Chem (2008) 283(9):5669–76. 10.1074/jbc.M708118200 18086673

[60] Violin JD Lefkowitz RJ. Beta-Arrestin-Biased Ligands at Seven-Transmembrane Receptors. Trends Pharmacol Sci (2007) 28(8):416–22. 10.1016/j.tips.2007.06.006 17644195

[61] Smith JS Lefkowitz RJ Rajagopal S. Biased Signalling: From Simple Switches to Allosteric Microprocessors. Nat Rev Drug Discov (2018) 17(4):243–60. 10.1038/nrd.2017.229 PMC5936084 29302067

[62] van Gastel J Hendrickx JO Leysen H Santos-Otte P Luttrell LM Martin B. Beta-Arrestin Based Receptor Signaling Paradigms: Potential Therapeutic Targets for Complex Age-Related Disorders. Front Pharmacol (2018) 9:1369. 10.3389/fphar.2018.01369 30546309 PMC6280185

[63] Woo AY Ge XY Pan L Xing G Mo YM Xing RJ. Discovery of Beta-Arrestin-Biased Beta2-Adrenoceptor Agonists From 2-Amino-2-Phenylethanol Derivatives. Acta Pharmacol Sin (2019) 40(8):1095–105. 10.1038/s41401-018-0200-x PMC6786399 30643208

[64] Ahn S Shenoy SK Wei H Lefkowitz RJ. Differential Kinetic and Spatial Patterns of Beta-Arrestin and G Protein-Mediated ERK Activation by the Angiotensin II Receptor. J Biol Chem (2004) 279(34):35518–25. 10.1074/jbc.M405878200 15205453

[65] Alvarez-Curto E Inoue A Jenkins L Raihan SZ Prihandoko R Tobin AB. Targeted Elimination of G Proteins and Arrestins Defines Their Specific Contributions to Both Intensity and Duration of G Protein-Coupled Receptor Signaling. J Biol Chem (2016) 291(53):27147–59. 10.1074/jbc.M116.754887 PMC5207144 27852822

[66] Caron A Reynolds RP Castorena CM Michael NJ Lee CE Lee S. Adipocyte Gs But Not Gi Signaling Regulates Whole-Body Glucose Homeostasis. Mol Metab (2019) 27:11–21. 10.1016/j.molmet.2019.06.019 31279640 PMC6717754

[67] Långberg EC Seed Ahmed M Efendic S Gu HF Östenson CG. Genetic Association of Adrenergic Receptor Alpha 2A With Obesity and Type 2 Diabetes. Obesity (2013) 21(8):1720–5. 10.1002/oby.20162 23526671

[68] Greenberg AS Egan JJ Wek SA Garty NB Blanchette-Mackie EJ Londos C. Perilipin, a Major Hormonally Regulated Adipocyte-Specific Phosphoprotein Associated With the Periphery of Lipid Storage Droplets. J Biol Chem (1991) 266(17):11341–6. 10.1016/S0021-9258(18)99168-4 2040638

[69] Priyadarshini M Villa SR Fuller M Wicksteed B Mackay CR Alquier T. FFAR2, Regulates Insulin Secretion. Mol Endocrinol (2015) 29(7):1055–66. 10.1210/me.2015-1007 PMC4484778 26075576

[70] Sebastiani G Ceccarelli E Castagna MG Dotta F. G-Protein-Coupled Receptors (GPCRs) in the Treatment of Diabetes: Current View and Future Perspectives. Best Pract Res Clin Endocrinol Metab (2018) 32(2):201–13. 10.1016/j.beem.2018.02.005 29678286

[71] O’Dowd BF Nguyen T Marchese A Cheng R Lynch KR Heng HH. Discovery of Three Novel G-Protein-Coupled Receptor Genes. Genomics (1998) 47(2):310–3. 10.1006/geno.1998.5095 9479505

[72] Wang J Simonavicius N Wu X Swaminath G Reagan J Tian H. Kynurenic Acid as a Ligand for Orphan G Protein-Coupled Receptor GPR35. J Biol Chem (2006) 281(31):22021–8. 10.1074/jbc.M603503200 16754668

[73] Ohshiro H Tonai-Kachi H Ichikawa K. GPR35 Is a Functional Receptor in Rat Dorsal Root Ganglion Neurons. Biochem Biophys Res Commun (2008) 365(2):344–8. 10.1016/j.bbrc.2007.10.197 17996730

[74] Okumura S Baba H Kumada T Nanmoku K Nakajima H Nakane Y. Cloning of a G-Protein-Coupled Receptor That Shows an Activity to Transform NIH3T3 Cells and Is Expressed in Gastric Cancer Cells. Cancer Sci (2004) 95(2):131–5. 10.1111/j.1349-7006.2004.tb03193.x PMC11159784 14965362

[75] Egerod KL Petersen N Timshel PN Rekling JC Wang Y Liu Q. Profiling of G Protein-Coupled Receptors in Vagal Afferents Reveals Novel Gut-to-Brain Sensing Mechanisms. Mol Metab (2018) 12:62–75. 10.1016/j.molmet.2018.03.016 29673577 PMC6001940

[76] Husted AS Trauelsen M Rudenko O Hjorth SA Schwartz TW. GPCR-Mediated Signaling of Metabolites. Cell Metab (2017) 25(4):777–96. 10.1016/j.cmet.2017.03.008 28380372

[77] Binti Mohd Amir NAS Mackenzie AE Jenkins L Boustani K Hillier MC Tsuchiya T. Evidence for the Existence of a CXCL17 Receptor Distinct From GPR35. J Immunol (Baltimore Md: 1950) (2018) 201(2):714–24. 10.4049/jimmunol.1700884 PMC6036231 29875152

[78] Park SJ Lee SJ Nam SY Im DS. GPR35 Mediates Lodoxamide-Induced Migration Inhibitory Response But Not CXCL17-Induced Migration Stimulatory Response in THP-1 Cells; Is GPR35 a Receptor for CXCL17? Br J Pharmacol (2018) 175(1):154–61. 10.1111/bph.14082 PMC5740256 29068046

[79] Itoh Y Kawamata Y Harada M Kobayashi M Fujii R Fukusumi S. Free Fatty Acids Regulate Insulin Secretion From Pancreatic β Cells Through GPR40. Nature (2003) 422(6928):173–6. 10.1038/nature01478 12629551

[80] Feng DD Luo Z S-g R Hernandez M Tawadros N DJ K. Reduction in Voltage-Gated K+ Currents in Primary Cultured Rat Pancreatic β-Cells by Linoleic Acids. Endocrinology (2006) 147(2):674–82. 10.1210/en.2005-0225 16254037

[81] Kim M Gu GJ Koh YS Lee SH Na YR Seok SH. Fasiglifam (TAK-875), a G Protein-Coupled Receptor 40 (GPR40) Agonist, May Induce Hepatotoxicity Through Reactive Oxygen Species Generation in a GPR40-Dependent Manner. Biomolecules Ther (2018) 26(6):599–607. 10.4062/biomolther.2017.225 PMC6254646 29429148

[82] Tolhurst G Heffron H Lam YS Parker HE Habib AM Diakogiannaki E. Short-Chain Fatty Acids Stimulate Glucagon-Like Peptide-1 Secretion *via* the G-Protein–Coupled Receptor Ffar2. Diabetes (2012) 61(2):364–71. 10.2337/db11-1019 PMC3266401 22190648

[83] Zaibi MS Stocker CJ O’Dowd J Davies A Bellahcene M Cawthorne MA. Roles of GPR41 and GPR43 in Leptin Secretory Responses of Murine Adipocytes to Short Chain Fatty Acids. FEBS Lett (2010) 584(11):2381–6. 10.1016/j.febslet.2010.04.027 20399779

[84] Tang C Ahmed K Gille A Lu S Gröne H-J Tunaru S. Loss of FFA2 and FFA3 Increases Insulin Secretion and Improves Glucose Tolerance in Type 2 Diabetes. Nat Med (2015) 21(2):173–7. 10.1038/nm.3779 25581519

[85] Nilsson NE Kotarsky K Owman C Olde B. Identification of a Free Fatty Acid Receptor, FFA2R, Expressed on Leukocytes and Activated by Short-Chain Fatty Acids. Biochem Biophys Res Commun (2003) 303(4):1047–52. 10.1016/S0006-291X(03)00488-1 12684041

[86] Senga T Iwamoto S Yoshida T Yokota T Adachi K Azuma E. LSSIG Is a Novel Murine Leukocyte-Specific GPCR That Is Induced by the Activation of STAT3. Blood J Am Soc Hematol (2003) 101(3):1185–7. 10.1182/blood-2002-06-1881 12393494

[87] Ang Z Er JZ Ding JL. The Short-Chain Fatty Acid Receptor GPR43 Is Transcriptionally Regulated by XBP1 in Human Monocytes. Sci Rep (2015) 5(1):1–9. 10.1038/srep08134 PMC4311239 25633224

[88] Lorza-Gil E Kaiser G Rexen Ulven E König GM Gerst F Oquendo MB. FFA2-, But Not FFA3-Agonists Inhibit GSIS of Human Pseudoislets: A Comparative Study With Mouse Islets and Rat INS-1E Cells. Sci Rep (2020) 10(1):16497. 10.1038/s41598-020-73467-5 33020504 PMC7536384

[89] Davidsson Ö Nilsson K Brånalt J Andersson T Berggren K Chen Y. Identification of Novel GPR81 Agonist Lead Series for Target Biology Evaluation. Bioorganic Medicinal Chem Lett (2020) 30(4):126953. 10.1016/j.bmcl.2020.126953 31932225

[90] Ahmed K Tunaru S Tang C Müller M Gille A Sassmann A. An Autocrine Lactate Loop Mediates Insulin-Dependent Inhibition of Lipolysis Through GPR81. Cell Metab (2010) 11(4):311–9. 10.1016/j.cmet.2010.02.012 20374963

[91] Wanders D Graff EC Judd RL. Effects of High Fat Diet on GPR109A and GPR81 Gene Expression. Biochem Biophys Res Commun (2012) 425(2):278–83. 10.1016/j.bbrc.2012.07.082 22842580

[92] Overton HA Babbs AJ Doel SM Fyfe MC Gardner LS Griffin G. Deorphanization of a G Protein-Coupled Receptor for Oleoylethanolamide and Its Use in the Discovery of Small-Molecule Hypophagic Agents. Cell Metab (2006) 3(3):167–75. 10.1016/j.cmet.2006.02.004 16517404

[93] Soga T Ohishi T Matsui T Saito T Matsumoto M Takasaki J. Lysophosphatidylcholine Enhances Glucose-Dependent Insulin Secretion *via* an Orphan G-Protein-Coupled Receptor. Biochem Biophys Res Commun (2005) 326(4):744–51. 10.1016/j.bbrc.2004.11.120 15607732

[94] Lan H Lin H Wang C Wright M Xu S Kang L. Agonists at GPR119 Mediate Secretion of GLP-1 From Mouse Enteroendocrine Cells Through Glucose-Independent Pathways. Br J Pharmacol (2012) 165(8):2799–807. 10.1111/j.1476-5381.2011.01754.x PMC3423251 22029751

[95] Abdel-Magid AF. GPR119 Modulators for the Treatment of Diabetes, Obesity, and Related Diseases: Patent Highlight. ACS Med Chem Lett (2012) 3(12):955–8. 10.1021/ml300296q PMC4025843 24900416

[96] Stone VM Dhayal S Brocklehurst KJ Lenaghan C Winzell MS Hammar M. GPR120 (FFAR4) Is Preferentially Expressed in Pancreatic Delta Cells and Regulates Somatostatin Secretion From Murine Islets of Langerhans. Diabetologia (2014) 57(6):1182–91. 10.1007/s00125-014-3213-0 PMC4018485 24663807

[97] Moore K Zhang Q Murgolo N Hosted T Duffy R. Cloning, Expression, and Pharmacological Characterization of the GPR120 Free Fatty Acid Receptor From Cynomolgus Monkey: Comparison With Human GPR120 Splice Variants. Comp Biochem Physiol Part B: Biochem Mol Biol (2009) 154(4):419–26. 10.1016/j.cbpb.2009.08.005 19723586

[98] Talukdar S Bae EJ Imamura T Morinaga H Fan W Li P. GPR120 Is an Omega-3 Fatty Acid Receptor Mediating Potent Anti-Inflammatory and Insulin-Sensitizing Effects. Cell (2010) 142(5):687–98. 10.1016/j.cell.2010.07.041 PMC2956412 20813258

[99] Villegas-Comonfort S Takei Y Tsujimoto G Hirasawa A García-Sáinz J. Effects of Arachidonic Acid on FFA4 Receptor: Signaling, Phosphorylation and Internalization. Prostaglandins Leukotrienes Essential Fatty Acids (2017) 117:1–10. 10.1016/j.plefa.2017.01.013 28237082

[100] Milligan G Alvarez-Curto E Watterson K Ulven T Hudson B. Characterizing Pharmacological Ligands to Study the Long-Chain Fatty Acid Receptors GPR 40/FFA 1 and GPR 120/FFA 4. Br J Pharmacol (2015) 172(13):3254–65. 10.1111/bph.12879 PMC4500364 25131623

[101] Li A Yang D Zhu M Tsai KC Xiao KH Yu X. Discovery of Novel FFA4 (GPR120) Receptor Agonists With β-Arrestin2-Biased Characteristics. Future Medicinal Chem (2015) 7(18):2429–37. 10.4155/fmc.15.160 26653412

[102] Fletcher Madeleine M Halls Michelle L Christopoulos A Sexton Patrick M Wootten D. The Complexity of Signalling Mediated by the Glucagon-Like Peptide-1 Receptor. Biochem Soc Trans (2016) 44(2):582–8. 10.1042/BST20150244 27068973

[103] Farilla L Bulotta A Hirshberg B Li Calzi S Khoury N Noushmehr H. Glucagon-Like Peptide 1 Inhibits Cell Apoptosis and Improves Glucose Responsiveness of Freshly Isolated Human Islets. Endocrinology (2003) 144(12):5149–58. 10.1210/en.2003-0323 12960095

[104] Kuna RS Girada SB Asalla S Vallentyne J Maddika S Patterson JT. Glucagon-Like Peptide-1 Receptor-Mediated Endosomal cAMP Generation Promotes Glucose-Stimulated Insulin Secretion in Pancreatic β-Cells. Am J Physiol Endocrinol Metab (2013) 305(2):E161–70. 10.1152/ajpendo.00551.2012 23592482

[105] Rajan S Dickson LM Mathew E Orr CM Ellenbroek JH Philipson LH. Chronic Hyperglycemia Downregulates GLP-1 Receptor Signaling in Pancreatic β-Cells *via* Protein Kinase A. Mol Metab (2015) 4(4):265–76. 10.1016/j.molmet.2015.01.010 PMC4354925 25830090

[106] Pabreja K Mohd MA Koole C Wootten D Furness SG. Molecular Mechanisms Underlying Physiological and Receptor Pleiotropic Effects Mediated by GLP-1R Activation. Br J Pharmacol (2014) 171(5):1114–28. 10.1111/bph.12313 PMC3952792 23889512

[107] Weston C Poyner D Patel V Dowell S Ladds G. Investigating G Protein Signalling Bias at the Glucagon-Like Peptide-1 Receptor in Yeast. Br J Pharmacol (2014) 171(15):3651–65. 10.1111/bph.12716 PMC4128063 24712679

[108] Jorgensen R Kubale V Vrecl M Schwartz TW Elling CE. Oxyntomodulin Differentially Affects Glucagon-Like Peptide-1 Receptor β-Arrestin Recruitment and Signaling Through Gα. J Pharmacol Exp Ther (2007) 322(1):148–54. 10.1124/jpet.107.120006 17395766

[109] Shukla AK Westfield GH Xiao K Reis RI Huang LY Tripathi-Shukla P. Visualization of Arrestin Recruitment by a G-Protein-Coupled Receptor. Nature (2014) 512(7513):218–22. 10.1038/nature13430 PMC4134437 25043026

[110] Thomsen ARB Plouffe B Cahill TJ 3rd Shukla AK Tarrasch JT Dosey AM. GPCR-G Protein-Beta-Arrestin Super-Complex Mediates Sustained G Protein Signaling. Cell (2016) 166(4):907–19. 10.1016/j.cell.2016.07.004 PMC5418658 27499021

[111] Zhuang LN Hu WX Zhang ML Xin SM Jia WP Zhao J. Beta-Arrestin-1 Protein Represses Diet-Induced Obesity. J Biol Chem (2011) 286(32):28396–402. 10.1074/jbc.M111.223206 PMC3151082 21543334

[112] Pydi SP Cui Z He Z Barella LF Pham J Cui Y. Beneficial Metabolic Role of β-Arrestin-1 Expressed by AgRP Neurons. Sci Adv (2020) 6(23):eaaz1341. 10.1126/sciadv.aaz1341 32537493 PMC7269658

[113] Pydi SP Jain S Tung W Cui Y Zhu L Sakamoto W. Adipocyte β-Arrestin-2 Is Essential for Maintaining Whole Body Glucose and Energy Homeostasis. Nat Commun (2019) 10(1):2936. 10.1038/s41467-019-11003-4 31270323 PMC6610117

[114] Zhu L Rossi M Cui Y Lee RJ Sakamoto W Perry NA. Hepatic β-Arrestin 2 Is Essential for Maintaining Euglycemia. J Clin Invest (2017) 127(8):2941–5. 10.1172/JCI92913 PMC5531395 28650340

[115] Barella LF Rossi M Zhu L Cui Y Mei FC Cheng X. β-Cell-Intrinsic β-Arrestin 1 Signaling Enhances Sulfonylurea-Induced Insulin Secretion. J Clin Invest (2019) 129(9):3732–7. 10.1172/JCI126309 PMC6715363 31184597

[116] Zhu L Almaça J Dadi PK Hong H Sakamoto W Rossi M. β-Arrestin-2 Is an Essential Regulator of Pancreatic β-Cell Function Under Physiological and Pathophysiological Conditions. Nat Commun (2017) 8(1):14295. 10.1038/ncomms14295 28145434 PMC5296650

[117] Meister J Bone DBJ Godlewski G Liu Z Lee RJ Vishnivetskiy SA. Metabolic Effects of Skeletal Muscle-Specific Deletion of Beta-Arrestin-1 and -2 in Mice. PloS Genet (2019) 15(10):e1008424. 10.1371/journal.pgen.1008424 31622341 PMC6818801

[118] Pydi SP Jain S Tung W Cui Y Zhu L Sakamoto W. Adipocyte Beta-Arrestin-2 Is Essential for Maintaining Whole Body Glucose and Energy Homeostasis. Nat Commun (2019) 10(1):2936. 10.1038/s41467-019-11003-4 31270323 PMC6610117

[119] Wang L Pydi SP Cui Y Zhu L Meister J Gavrilova O. Selective Activation of Gs Signaling in Adipocytes Causes Striking Metabolic Improvements in Mice. Mol Metab (2019) 27:83–91. 10.1016/j.molmet.2019.06.018 31272886 PMC6717953

[120] Pydi SP Jain S Barella LF Zhu L Sakamoto W Meister J. β-Arrestin-1 Suppresses Myogenic Reprogramming of Brown Fat to Maintain Euglycemia. Sci Advances (2020) 6(23):eaba1733. 10.1126/sciadv.aba1733 32548266 PMC7274797

[121] Dalle S Imamura T Rose DW Worrall DS Ugi S Hupfeld CJ. Insulin Induces Heterologous Desensitization of G-Protein-Coupled Receptor and Insulin-Like Growth Factor I Signaling by Downregulating Beta-Arrestin-1. Mol Cell Biol (2002) 22(17):6272–85. 10.1128/MCB.22.17.6272-6285.2002 PMC134007 12167719

[122] Usui I Imamura T Huang J Satoh H Shenoy SK Lefkowitz RJ. β-Arrestin-1 Competitively Inhibits Insulin-Induced Ubiquitination and Degradation of Insulin Receptor Substrate 1. Mol Cell Biol (2004) 24(20):8929–37. 10.1128/MCB.24.20.8929-8937.2004 PMC517874 15456867

[123] Pocai A Lam TK Gutierrez-Juarez R Obici S Schwartz GJ Bryan J. Hypothalamic K(ATP) Channels Control Hepatic Glucose Production. Nature (2005) 434(7036):1026–31. 10.1038/nature03439 15846348

[124] Gropp E Shanabrough M Borok E Xu AW Janoschek R Buch T. Agouti-Related Peptide-Expressing Neurons Are Mandatory for Feeding. Nat Neurosci (2005) 8(10):1289–91. 10.1038/nn1548 16158063

[125] Luquet S Perez FA Hnasko TS Palmiter RD. NPY/AgRP Neurons Are Essential for Feeding in Adult Mice But can be Ablated in Neonates. Science (2005) 310(5748):683–5. 10.1126/science.1115524 16254186

[126] Spanswick D Smith MA Groppi VE Logan SD Ashford ML. Leptin Inhibits Hypothalamic Neurons by Activation of ATP-Sensitive Potassium Channels. Nature (1997) 390(6659):521–5. 10.1038/37379 9394003

[127] Spanswick D Smith MA Mirshamsi S Routh VH Ashford ML. Insulin Activates ATP-Sensitive K+ Channels in Hypothalamic Neurons of Lean, But Not Obese Rats. Nat Neurosci (2000) 3(8):757–8. 10.1038/77660 10903566

[128] Lin HV Accili D. Hormonal Regulation of Hepatic Glucose Production in Health and Disease. Cell Metab (2011) 14(1):9–19. 10.1016/j.cmet.2011.06.003 21723500 PMC3131084

[129] Cho YM Merchant CE Kieffer TJ. Targeting the Glucagon Receptor Family for Diabetes and Obesity Therapy. Pharmacol Ther (2012) 135(3):247–78. 10.1016/j.pharmthera.2012.05.009 22659620

[130] Htike ZZ Zaccardi F Papamargaritis D Webb DR Khunti K Davies MJ. Efficacy and Safety of Glucagon-Like Peptide-1 Receptor Agonists in Type 2 Diabetes: A Systematic Review and Mixed-Treatment Comparison Analysis. Diabetes Obes Metab (2017) 19(4):524–36. 10.1111/dom.12849 27981757

[131] Zhu L Rossi M Cui Y Lee RJ Sakamoto W Perry NA. Hepatic Beta-Arrestin 2 Is Essential for Maintaining Euglycemia. J Clin Invest (2017) 127(8):2941–5. 10.1172/JCI92913 PMC5531395 28650340

